# Employing Eye Trackers to Reduce Nuisance Alarms

**DOI:** 10.3390/s25092635

**Published:** 2025-04-22

**Authors:** Katherine Herdt, Michael Hildebrandt, Katya LeBlanc, Nathan Lau

**Affiliations:** 1 Grado Department of Industrial and Systems Engineering, Virginia Tech, Blacksburg, VA 24061, USA; katherine.herdt1@gevernova.us; 2 Humans and Automation Department, Institute for Energy Technology, 1777 Halden, Norway; michael.hildebrandt@ife.no; 3 Department of Human Factors and Reliability, Idaho National Laboratory, Idaho Falls, ID 83415, USA; katya.leblanc@inl.gov

**Keywords:** eye tracking, alarms, attention, user interface, monitoring

## Abstract

When process operators anticipate an alarm prior to its annunciation, that alarm loses information value and becomes a nuisance. This study investigated using eye trackers to measure and adjust the salience of alarms with three methods of gaze-based acknowledgement (GBA) of alarms that estimate operator anticipation. When these methods detected possible alarm anticipation, the alarm’s audio and visual salience was reduced. A total of 24 engineering students (male = 14, female = 10) aged between 18 and 45 were recruited to predict alarms and control a process parameter in three scenario types (parameter near threshold, trending, or fluctuating). The study evaluated whether behaviors of the monitored parameter affected how frequently the three GBA methods were utilized and whether reducing alarm salience improved control task performance. The results did not show significant task improvement with any GBA methods (F(3,69) = 1.357, *p* = 0.263, partial η^2^ = 0.056). However, the scenario type affected which GBA method was more utilized (*X*^2^ (2, *N* = 432) = 30.147, *p <* 0.001). Alarm prediction hits with gaze-based acknowledgements coincided more frequently than alarm prediction hits without gaze-based acknowledgements (*X*^2^ (1, *N* = 432) = 23.802, *p* < 0.001, OR = 3.877, 95% CI 2.25–6.68, *p* < 0.05). Participant ratings indicated an overall preference for the three GBA methods over a standard alarm design (F(3,63) = 3.745, *p* = 0.015, partial η^2^ = 0.151). This study provides empirical evidence for the potential of eye tracking in alarm management but highlights the need for additional research to increase validity for inferring alarm anticipation.

## 1. Introduction

Alarms serve as “an audible and/or visible means of indicating […] equipment malfunction, process deviation, or abnormal condition requiring a response” ([1] p. 14; [2]). Operators can only monitor process indicators selectively amongst other irrelevant indicators or “noise” in search of unexpected indications and abnormal situations [3,4]. Thus, alarms are a means of shifting the operator’s attention towards unnoticed parameters [5] by exploiting the person’s instinctive detection of light and motion in their peripheral vision and omnidirectional perception of sound [6,7,8]. The sound instantly alerts the operator and overrides concentration on ongoing visual tasks [9,10] and then prompts a search for the flashing light to pinpoint the location of visual information.

This saliency (or the attention attracting display features) can be problematic when alarms inappropriately redirect an operator’s focused attention [11]. Alarms can mistakenly convey an emergency when the crisis does not occur (false alarms) or become an annoyance from context insensitivity (nuisance alarms [12]). Nuisance alarms are commonly caused by (1) tightly coupled equipment [13], (2) small fluctuations [14], or (3) non-safety information [15]. From a signal detection perspective [13], current alarm implementation across domains tends to produce excessive false positives to avoid the consequences of having false negatives, leading to alarm fatigue and workload (e.g., [14,15]). Collectively, nuisance alarms can overwhelm operators because “many nearly simultaneous alarms [have] varying degrees of relevance to the operators’ tasks”, which constantly requires them to decide if the alarm is worth attending to ([16], p. 4-2).

### 1.1. Two Complementary Approaches to Advanced Alarm Management

Research has taken two general approaches to alleviate excessive nuisance alarms: process-centric and user-centric alarm management. Process-centric alarm management relies on measuring plant process behaviors and observing system characteristics to verify alarm value. Event correlation analysis and multivariate process monitoring reduce alarms that frequently occur together in process data [17,18,19,20], allowing engineers to group alarms together without removing any information about the plant’s state. Rodrigo et al. [21] and Hu et al. [22] employed a similarity index with transfer entropy to reveal that two main parameter alarms would suffice during a 3-day alarm flood. Employing dynamic fault trees decreased alarms by 80% on helicopter flights [23]. Time delays between events or a multi-temporal sequence algorithm can reveal alarm causality for a chemical plant incident and a water treatment testbed, respectively [24,25]. Further, “chattering alarms” caused by normal fluctuations can be minimized by combining time-delays, filters, and dead-bands on process variables to optimize false alarms and misses based on the Receiver Operating Characteristic (ROC) curve [2,26,27].

State-based alarms employ thresholds specific to operational modes [15,22,28]. Likewise, model-based plant monitoring compares the expected plant response to actual plant behavior to verify true emergencies [18,29]. In healthcare, where alarm fatigue is common [30], researchers have investigated state-based (or patient-based) alarms that personalize alarm thresholds based on a patient’s first day vital signs to provide a more appropriate baseline [31]. van Rossum et al. [32] investigated six adaptive threshold-based alarm strategies to account for patient characteristics and situational factors, which revealed adaptive alarm strategies to be most effective at increasing sensitivity while minimizing alarms. Collectively, these process-centric techniques can greatly reduce nuisance alarms.

Alternatively, user-centric alarm management manages alarms based on how the operator evaluates the situation. This approach includes having operators prescribe temporary alarms, adjust setpoints, configure filter options, and sort alarms as needed [16,28,33,34], thereby allowing end users to finish the design (supported by the ecological approach; see [35,36]). However, giving operators the ability to alter setpoints sometimes increases missed events and places an additional workload of reconfiguring alarms [37,38,39]. The latest research on user monitoring and adaptive user interfaces has utilized unobtrusive sensors to sound alarms. For example, electrocardiogram, electrodermal activity, and gaze-based physiological metrics are being used to monitor driver workload, fatigue, and attention [40,41,42] to alert drivers of impending safety problems or even crashes [43]. However, such an approach has not been adopted in control rooms of processing plants.

While a false alarm is based on the validity of an event, a nuisance alarm is based on an operator’s perceived value of the information. Positive Predictive Value (PPV), a measure of alarm informativeness, decreases with operator vigilance due to greater anticipation [44,45,46,47] because expected alarms lack novel information and become a burden for the operators to acknowledge manually. Typical alarm configurations and process-centric alarm management do not account for operator’s expectancy, leading to various situations in which these nuisance alarms occur [12]. The premise of an alarm is to provide monitoring backup that should limit alerts on information unknown to the operator. Therefore, complete removal of nuisance alarms cannot occur without considering real-time monitoring behaviors that affects operator event expectancy or situation awareness [3,48].

There are scarce investigations into operator monitoring performance to adapt alarms in real time. Niwa and Hollnagel [49] proposed modulating alarm presentation based on real-time assessment of an operator’s control of the plant. In cases where low-level control is detected, alarm presentation is simplified to give an overview. Using the Tennessee Eastman process simulation, Li, Wang, and Yang [50] demonstrated a reduction in alarms with their adaptive optimization for the alarm’s threshold based on operator perceptual capacity and the ratio of untreated alarms to the total number of alarms. Neither study implemented nor assessed their designs. Despite the proliferation of biometric sensors [51], there is no recent methodological or empirical research on the real-time estimation of operator expectancy to support user-centric alarm management.

### 1.2. Eye Movements and Parameter Behaviors for Alarm Management

Eye tracking could provide a strong basis for user-centric alarm management by characterizing top–down and reflex-driven visual perception that reflects the ongoing cognitive processes of operators in real time [52]. As the control room displays rely mainly on the visual modality, the eye tracker is thus a highly compatible sensor for measuring what system information the operator is attending to (i.e., compatibility between sensor and display modality). In addition, theories in different subdisciplines of psychology have received empirical support demonstrating that eye metrics are good correlates for gauging awareness and performance [53]. From foundational psychology, the information reduction theory and the eye–mind hypothesis indicate that the gaze can reflect what the operator is thinking about [54,55]; long-term memory research indicates that the gaze can reflect the retrieval of knowledge for decision making and actions relevant to specific information (e.g., [53,56]); and expertise research indicates that the gaze can reflect the level of cognitive processing regarding specific information (e.g., [53,57]). Furthermore, eye tracking provides non-intrusive, real-time data that does not impose additional workload on the operators by requiring them to verbalize their thoughts and/or click on parameters of interests. In other words, the eye tracker serves as both a non-intrusive and compatible sensor for measuring visual attention.

Applied research has employed eye metrics to evaluate overview displays [58], estimate cognitive states [59], assess training progress [60,61], measure fatigue [62], and infer SA [63] in control room settings. Outside of the control room, look ahead eye fixations can reveal future hand movements in surgery and steering in driving [64,65,66]. While ample research indicates that gaze metrics could help infer operator awareness of information about the system on visual displays, the literature does not contain any studies employing eye metrics to infer operator expectancy for managing alarms, calling for further research on such applications and evaluation. For estimating operator anticipation of alarms, this work draws on some seminal research on process monitoring and eye movements that adjust to different process parameter behaviors. Parameters hovering near the control limit lead to increased visual sampling [67,68]. A field study revealed that operators establish parameter checkpoints that are more conservative than alarm thresholds. They watch these mental checkpoints closely to reach their performance goals [69,70]. In a field study of a nuclear power plant, Vicente et al. [71] observed that operators tightened alarm limits or created pre-alarms if they suspected drifting or troublesome parameters. These findings suggest that frequent fixations on a parameter near its control limit are likely correlated with alarm expectation.

Parameter fluctuation also influences monitoring behavior. Fluctuating parameters are less predictable, providing more information per visual sample (based on information theory [72]). Fixations are more frequent and gaze durations are higher on parameters with greater entropy [73]. Likewise, slope variability induces higher sampling rates compared to parameters changing at constant rates [74]. Thus, models of monitoring behaviors generally predict that operators wait to visually sample a parameter until the parameter entropy is sufficiently high to maximize the value of their effort [67,75,76]. Collectively, this suggests that increased sampling of fluctuating parameters may align with alarm anticipation.

Parameter trends inform operators of impending alarms. Control room displays adopt trend graphs to show patterns of past data for predicting future behavior [77]. In Robinson’s study [78], operator estimation of the future value of a dial was determined by the current value minus the average change observed, demonstrating that human estimation is systematic and relies on historical data. Similarly, one process monitoring model describes an operator’s estimation of parameter values as a probability distribution curve based on each visual sample [69]. Hence, a more consistent trendline increases the likelihood of a correct inference based on a narrower and more concentrated probability distribution curve. Fixation frequency is also positively correlated with the parameter’s rate of change [74,79]. These findings demonstrate that an operator has greater alarm expectancy with increasing steadiness and slope magnitude in the parameter trends.

Eye tracking can monitor operators’ visual sampling behaviors expected for different process parameter behaviors for inferring awareness and thus the information value of alarms. Thus, eye tracking can provide a means to relieve operators from manually acknowledging anticipated alarms that have low informative value and “attention-grabbing” visual and auditory signals that become a disruptive nuisance.

### 1.3. Overview of This Study

To evaluate this user-centric alarm management approach, we designed three gaze-based acknowledgement (GBA) methods that function based on the parameter behavior and the number of eye fixations on the impending alarm prior to annunciation. The methods differ based on the three types of parameter behaviors known to attract visual attention that were discussed in Section 1.2 and are intended to cater to such scenarios [80]. If the gaze-based methods estimate operator awareness of an alarm, the alarm is acknowledged automatically by reducing audio and visual saliency. The purpose of this study was to explore the extent to which eye fixations can correctly estimate operator awareness and anticipation of alarms and whether eye fixation can be used to moderate alarm salience for improving task performance (e.g., maintain steady states by keeping key process parameter in range). Specifically, we conducted a dual task experiment where the participant monitored alarms while simultaneously controlling a single parameter within a specified range. Each GBA method was tested across three scenario types that represent the different parameter behaviors known to attract visual attention (parameters near threshold, trending, or fluctuating). The research questions for this study are the following:

1. How do the GBA methods support parallel tasks of controlling process parameters and monitoring a set of parameters for alarms?
2. How does the compatibility between visual sampling and parameter behaviors affect the frequency of gaze acknowledgements for user-centric alarm management?

Answering these questions offers two contributions to science and engineering. First, this is the first study adopting such an innovative application of an eye tracker for user-centric alarm management, contributing the first empirical evidence in leveraging eye tracking sensors to design alarm systems for control rooms. The practical implications of extending eye tracking for real-time user monitoring and interaction design in industrial processing plants can improve safety and productivity. Second, the empirical findings can advance our understanding of how eye gaze data can infer awareness with respect to prior research on visual sampling in safety-critical systems. This can have implications for building a model-based approach to estimating and predicting cognitive states.

## 2. Materials and Methods

### 2.1. Participants

The study recruited 24 participants (male = 14, female = 10, of which 13 were 18–25 years old, 10 were 26–35, and 1 was 36–45) who were engineering students at a major university in Virginia. The sample size requirement was estimated to be twenty using G*Power 3.1 Software for a repeated measure ANOVA of four groups (i.e., acknowledgement methods) with three repeated measurements at an alpha value of 0.05 and beta value of 0.2 given a medium effect size f of 0.3 and correlation between repeated measures of 0.5.

The participants had little to no experience with industrial operations, and prerequisite knowledge of simulator plant operation was not an inclusion criterion. The GBA methods in this study did not account for the unique mental models and strategies of professional operators. Therefore, this research began investigation into the psychophysical aspects of the gaze interaction that can be tested without prior operational experience. The inclusion criteria for this study were non-vulnerable populations with normal or corrected-to-normal vision and no history of photosensitive epilepsy.

### 2.2. Experimental Apparatus and Tasks

**Figure 1:**
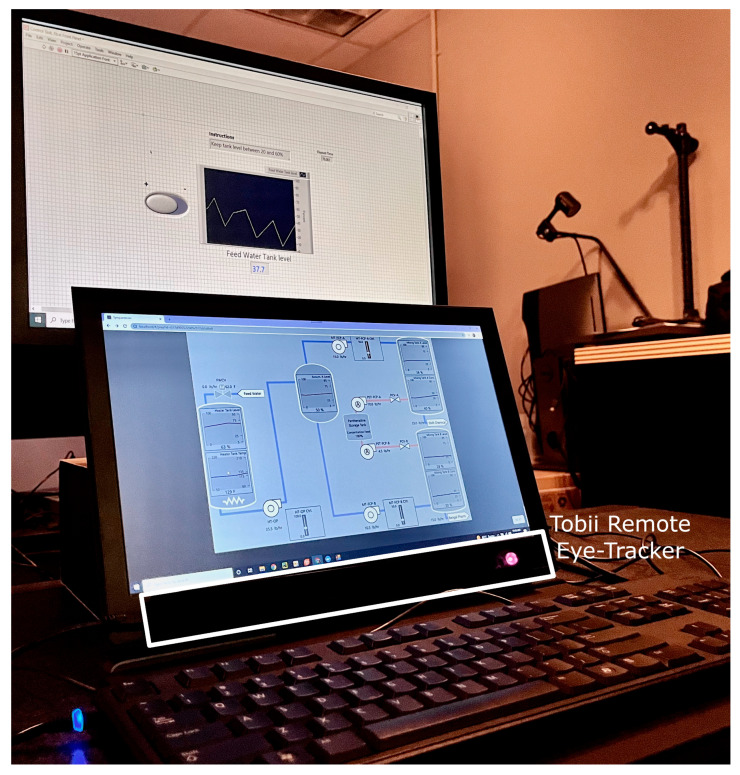
The experimental apparatus consisted of an LCD screen (top) for the participants to keep one parameter within 20–60% capacity by regulating a valve (top monitor) and another LCD screen (bottom) to monitor a set of parameters and predict any alarms in a chemical plant simulation.

The experimental apparatus (Figure 1) consisted of an ASUS ROG laptop computer with an Intel i7 6700 2.6 GhZ CPU processor and GTX 1060 GPU (ASUS, Taipei, Taiwan) connected to one external LCD monitor, a keyboard, and a mouse for multi-tasking. The bottom laptop monitor presented the primary task, where participants monitored for alarms. The Tobii X3-120 remote eye tracker (Tobii Technology, Stockholm, Sweden) was attached to the bottom monitor to collect eye gaze data. The top external monitor presented the secondary task, where participants controlled a single parameter.

**Figure 2:**
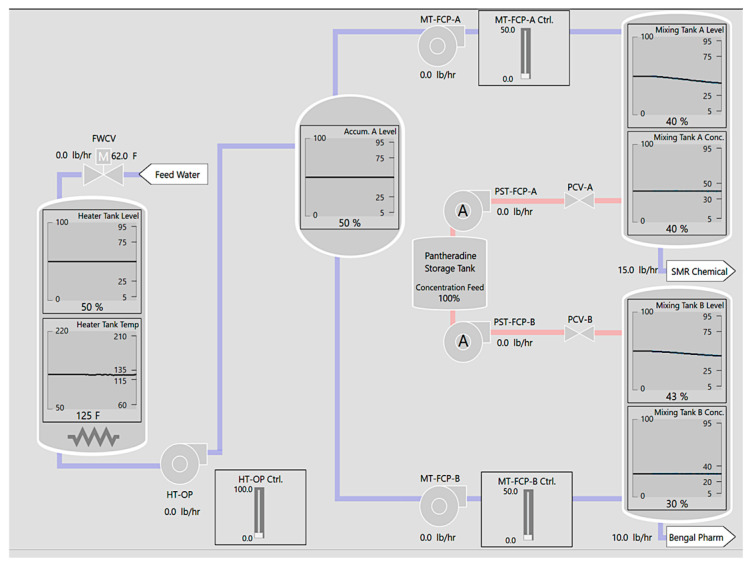
Chemical plant simulator used for the alarm monitoring task. The simulator has seven components that can alarm. Each parameter has marks noting the alarm set points and a trendline graph.

**Figure 3:**
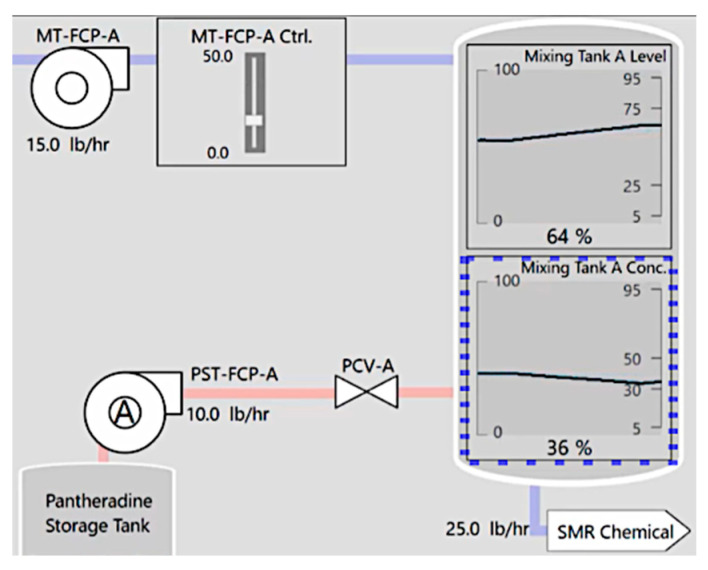
When participants click on a parameter, a blue dotted outline signifies the participant’s prediction of the impending alarm.

Participants monitored a set of parameters for alarms by observing video clips of simulated plant operations (Figure 2, [80]). Each parameter displayed a trendline of operation history over the last 15 s and marks showing the permissible operating range. Participants were asked to predict which parameter would alarm within four seconds by clicking on the parameter with their mouse. A blue dotted outline marked their prediction, which remained for three seconds (Figure 3). Simultaneously, Synquesticon [81], the custom server–client application hosted in Microsoft Azure that supports and executes ”microtasks”, computed the participants’ eye fixations on the AOI using the Velocity-Threshold Identification (I-VT) algorithm [82] in real time based on an angular velocity threshold of 30 degrees per second. Eye gaze was sampled at 60 Hz in batch sizes of 30.

**Figure 4:**
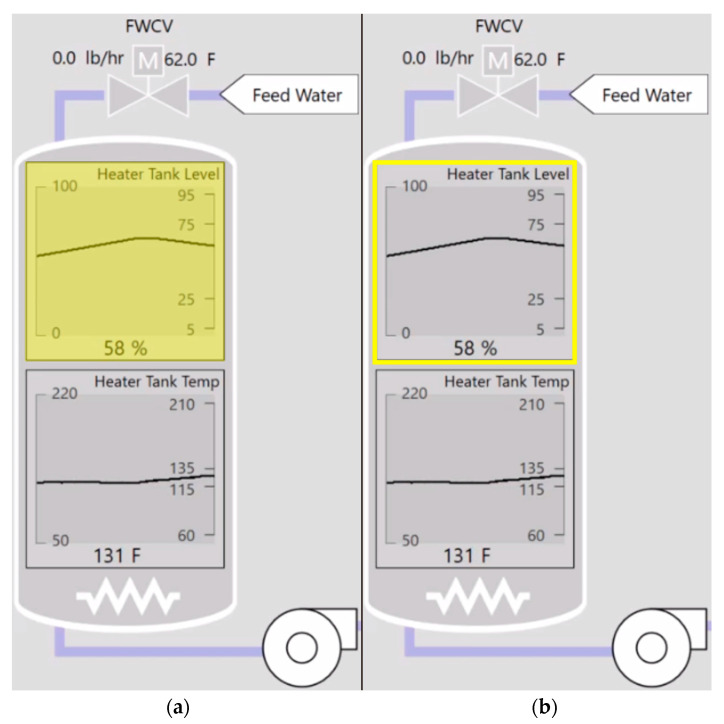
The original alarm presentation (**a**) versus an alarm during gaze-based acknowledgement (**b**).

When requirements for individual GBA methods were met (refer to Section 2.3.1), visual and auditory signals of the alarm were reduced to better match the level of alarm informativeness. Specifically, a normal alarm highlighted the parameter in yellow and produced a chime sound, while a gaze-based acknowledged alarm produced a yellow visual outline of the parameter and no sound (Figure 4). Reducing the visual cues was intended to stop peripheral vision from redirecting focal vision from the parameter control task. Muting audio was intended to minimize auditory preemption that would override attention towards the parameter control task or any ongoing visual tasks [9,10].

**Figure 5:**
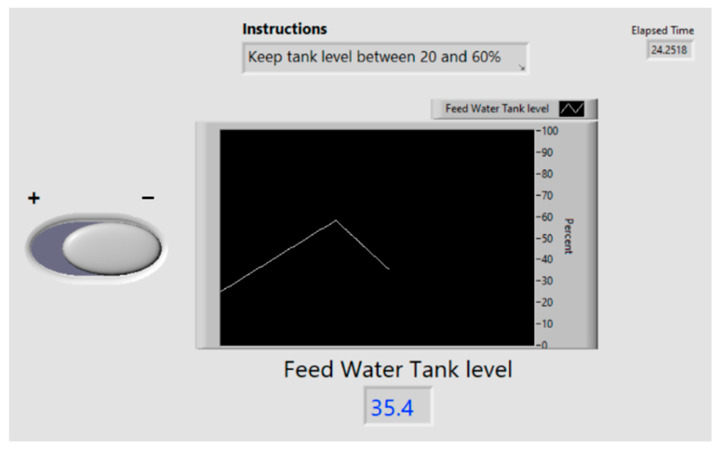
The parameter control task consisted of participants maintaining the feedwater tank level between 20 and 60% by toggling the valve on/off.

While monitoring the alarms, participants simultaneously controlled a process of maintaining the feedwater tank level between 20 and 60% in a LabView application. Participants were able to open a drain with a toggle switch on the user interface (Figure 5). If the level became too high (reaching 60% full), the participant could press “Enter” to open the drain, which caused the slope to decrease by 1.5 units every 100 ms. Equation (1) defines the process value behaviors of the feedwater tank level. Three versions of the parameter control task were randomly administered across trials for all participants. The versions varied in the starting value of the water tank level, *C* (25, 40, and 55%), and slope, *m*, varied randomly between 0.1 and 0.9 every 6 s. This was done to limit the learning effects of when to open the drain between trials.

The equation manipulating the feedwater tank level was designed to mandate at least one control action every 10 s (for the purpose of redirecting visual attention for this study rather than being representative of real-world operations).

Y = m(*t*) + C − 20(*d*) (1)

where Y is the current parameter value; m is the slope; *t* is the time from the start of the task; C is the starting value of the parameter; and *d* is the duration of time during which the drain is open.

### 2.3. Experimental Manipulations

There were two experimental manipulations: (1) the GBA method and (2) scenario types.

#### 2.3.1. Gaze-Based Acknowledgment (GBA) Method

The GBA methods minimize salience by altering the alarm to a visual outline and producing no audio when two criteria are satisfied. This study investigated three methods and a control condition that did not allow for acknowledgement. First, all three GBA methods acknowledged the alarm and altered its salience if two or more fixations were detected on the parameter that alarms in the scenario (i.e., areas of interest, AOI) within a time window of one to four seconds prior to the alarm. This time window was selected from research that showed a three to five second window being the most accurate for predicting intention [83].

**Figure 6:**
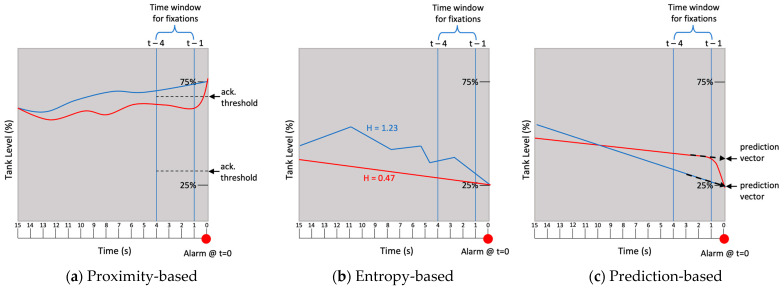
Graphical representations of (**a**) proximity-based GBA method, (**b**) entropy-based GBA method, and (**c**) prediction-based GBA method. The X-axis represents the time scale in seconds, and the y-axis represents the parameter scale in percentage. The blue line in each graph shows that acknowledgements muted some visual and auditory signals of the alarm at t = 0 if two fixations landed on the target parameters within the time window. The red line in each graph shows that acknowledgement did not occur regardless of the number of fixations on the target AOI within the time window for fixations because the parameter behaviors depicted by the red lines did not satisfy the pre-defined criteria.

Second, the three GBA methods only muted signals of the alarm if the following criteria were also satisfied:

1. The proximity-based method acknowledged an alarm when the parameter value exceeded the acknowledgement thresholds set at 10% and 90% of the range between high and low alarm setpoints. Figure 6a presents instances of a parameter activating (blue line) and not activating (red line) alarm acknowledgement through the proximity-based GBA method.
2. The entropy-based method acknowledged an alarm when the parameter values of the past 15 s window exceeded an entropy value of 1.2. Entropy was calculated using Equation (2) and a histogram estimator of the parameter value frequencies in 15 s batches, which represented the amount of history visible in the trendline graph [84,85]. The entropy threshold of 1.2 was determined specific for this simulator based on the maximum entropy that could be displayed on the parameter trend graph in the plant simulator. Maximum displayable entropy was a suitable choice for the initial assessment of GBA to minimize the risk of the participants being confused from not being able to discern small fluctuation on the display. Figure 6b shows a graphical representation of the entropy-based method.
  (2) $$H(X)=-\sum_{k=1}^{n}p_{k}log\left(\frac{p_{k}}{w_{k}}\right)$$
  where *n* is the number of intervals/bins, *k* is the interval/bin number, *p_k_* is the probability of *k*th interval/bin, and *w_k_* is the width of *k*th interval/bin.
3. The prediction-based method acknowledged an alarm when the predicted value of the parameter exceeded the alarm thresholds. The predicted value was calculated using a second-degree polynomial curve fitted to parameter values recorded over the past 15 s. The predicted value three seconds ahead of the simulator was compared to the actual value during the fixation window. Figure 6c shows a graphical representation of the prediction-based method.

While the principles guiding each GBA method are transferable across systems and domains, the exact thresholds for each method were designed specific to the plant simulator used for this study. The thresholds and prediction methods were based on pilot testing that could be considered equivalent to how control engineers examine historical data to redefine alarms or refine predictive control algorithms. These three GBA methods were compared to the fourth condition, where alarm presentation was unaltered regardless of gaze behaviors (i.e., fixations on the AOI at any time did not result in any acknowledgements of alarm). Representing current practice in alarm implementation, this condition served as a baseline comparison (Figure 4).

#### 2.3.2. Scenario Type

GBA methods were evaluated across three scenario types, which were defined by the parameter behavior of the target AOI/parameter right before the alarm. Each video scenario was 60 s long, containing one alarm. The target AOI varied evenly by the location on the LCD monitor (i.e., left, middle, right). The three scenario types were the following:

1. Near-threshold scenarios—a parameter stabilizing near the alarm threshold and thereby presenting an imminent risk of crossing the threshold.
2. Trending scenarios—a parameter increasing or decreasing towards the alarm thresholds continuously for four or more seconds and thereby presenting an increasing risk of crossing the alarm thresholds.
3. Fluctuation scenarios—a parameter having entropy greater than one based on the past 15 s of parameter values and thereby presenting a risk of crossing the threshold in an unexpected manner.

### 2.4. Experimental Design

This study employed a 4 × 3 fully crossed within-subject design with a treatment of GBA method of four levels (proximity-based, prediction-based, entropy-based, and no acknowledgement) and a treatment of scenario type of three levels (near threshold, trending, and fluctuation). The treatment of the GBA method specified the criteria to determine whether gaze acknowledgement of alarms occurred during the trials. The participants were administered each GBA method in a block of six trials. The presentation order of GBA methods was fully counterbalanced given the four conditions distributed over 24 participants (i.e., 4! = 24). There were two scenarios representing each scenario type. Scenario presentation order was randomized with no repeating permutations. The total number of trials was *N* = 576 (i.e., 24 participants × 4 acknowledgement methods × 3 scenario types × 2 scenarios of each type).

### 2.5. Procedure

Participants were emailed study details and gave verbal consent upon arrival. They received training on alarm monitoring and parameter control tasks through a PowerPoint presentation, followed by eye tracker calibration. Participants had two practice trials of the parameter prediction task and two practice trials of the parameter control task and then three practice trials of the tasks simultaneously. For each trial, the parameter control task started 10 s prior to the alarm monitoring task. Each trial only contained one alarm. The duration of each trial was 70 s long. They completed four blocks of six trials, with each block providing one GBA method. Providing a GBA method did not guarantee muting some visual and auditory signals of the alarm in the trial because the participant might not fixate at the parameter sufficiently between four and one seconds prior to the alarm. After each block, participants answered a short usability questionnaire. Then, a break was provided. Recalibration took place after each break. After completing four blocks, participants were debriefed and compensated USD 15. The study’s duration was approximately 90 min.

### 2.6. Measurements

Table 1 outlines the measurements collected during the study. Parameter control task performance was defined by the time out of range for the feedwater tank level, which quantified error and indicated how well the tank level was maintained within the prescribed thresholds. Alarm prediction performance was estimated by the number of prediction signal hits and misses. A prediction was counted as a hit for the trial when (1) the participant clicked on the parameter between four and one seconds before an alarm, and (2) the parameter selected was the target AOI (Figure 6). Otherwise, the prediction was counted as a signal miss for the trial. That is, if either the timing was off or the wrong parameter was selected, the prediction was deemed a miss. While the foundation for measuring alarm performance was based on signal detection theory (hit, miss, false alarm, correct rejection), each trial required monitoring multiple parameters as targets, and an alarm was always present in a trial. Thus, the experimental data were not suited to computing sensitivity and bias in signal detection theory because correct rejection and false alarm could result from either identifying (or clicking on) any one of the several parameters that did not cross the alarm thresholds or clicking too early. Table 2 describes alarm prediction performance in terms of signal detection theory for this study. Measuring alarm prediction hits and misses provided a way to verify how well alarm acknowledgements through fixation matched explicit alarm anticipation because eye gaze alone could not serve as both interaction and authentication of awareness for the experiment. GBA usage was defined by the number of alarm acknowledgements that did mute some visual and auditory signals of an alarm, thereby reducing its salience. Lastly, subjective usability of GBA methods was measured using a 3-item questionnaire about participants’ perception of the designs using 5-point Likert scales with anchors between “strongly agree” and “strongly disagree” (Table 3).

### 2.7. Hypotheses

The experimental hypotheses and their rationales are as follows.

1. A main effect that any GBA method would yield better parameter control task performance (less time out of range for the feedwater tank level; Table 1) than no acknowledgement because of the reduced salience associated with GBA and minimized distraction from the parameter control task.
2. A main effect that GBA methods would yield higher subjective usability ratings than no acknowledgement because the alarms would be less disruptive.
3. An interaction effect between GBA methods and scenario types on parameter control task performance because each GBA method was designed for specific parameter behaviors. In other words, there should be less time out of range for the feedwater tank level and more alarm acknowledgments (Table 1) when the parameter behavior of a scenario type matched the intended method. Specifically, it is predicted that the proximity-based method would yield the best performance in terms of the most alarm prediction hits and the least misses for near-threshold scenarios, the prediction-based method for trending scenarios, and the entropy-based method for fluctuation scenarios (Table 4).

## 3. Results

### 3.1. Analysis

A 4 × 3 repeated measures ANOVA with fixed factors of GBA method and scenario type was conducted to analyze control task performance or time out of range (with *N* = 288 given four acknowledgement methods, three scenario types, and 24 participants). Another repeated measures ANOVA with a fixed factor of GBA method was conducted to compare subjective usability ratings between three GBA methods (with *N* = 88 given four acknowledgement methods × 22 participants, as 2 participants did not complete the questionnaire properly). Significant effects are presented with means plots, which omit confidence intervals and corresponding discussion to avoid statistical inference with repeated measures ANOVA (refer to [86]).

Chi-squared tests were performed to test significant association (1) between alarm prediction hits/misses, GBA methods, and scenario types and (2) between alarm acknowledgement frequency (i.e., usage), GBA methods, and scenario types. Contingency tables are presented with any significant Chi-square tests.

### 3.2. Parameter Control Task Performance

**Figure 7:**
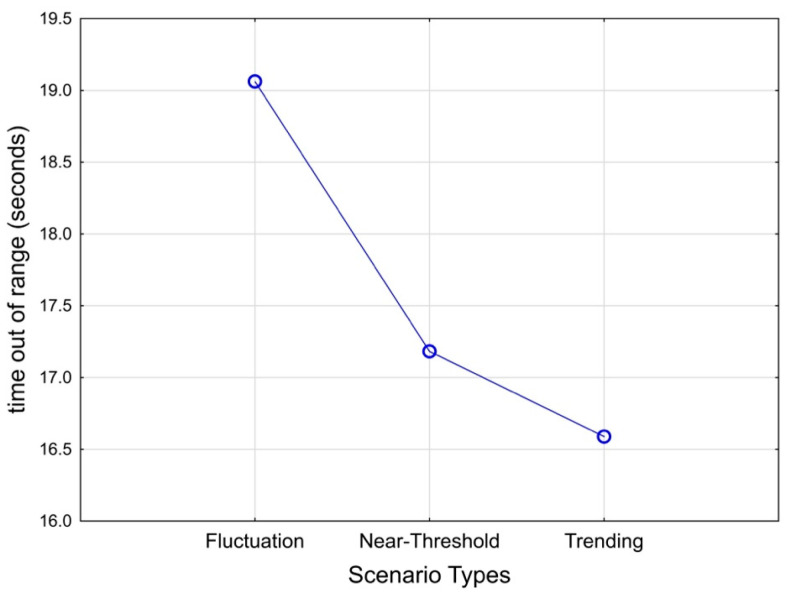
Mean plot of scenario type main effect on parameter control task performance, F(2,46) = 5.075, *p* = 0.011, partial η^2^ = 0.181.

The time out of range for the feedwater tank level did not have error residuals that were not normally distributed (Shapiro–Wilk test W = 0.981, *p* < 0.01), but the kurtosis and skewness were −0.56 and 0.33 within the range between 1 and −1, respectively; therefore, the ANOVA should remain robust against the normality assumption [87,88]. The ANOVA (Table 4) revealed a significant main effect of scenario type on time out of range for the parameter control task, F(2, 46) = 5.075, *p* = 0.011, partial η^2^ = 0.184. Fluctuation scenarios had the largest mean compared to near threshold or trending (Figure 7). Given the significance of the omnibus test without any a priori hypothesis regarding the differences between scenario conditions, post hoc tests adopting the Bonferroni adjustment to Type 1 error rate revealed that time out of range for fluctuation (M = 19.06, SE = 1.56) was significantly larger than that of trending scenarios (M = 16.60, SE = 1.54, *p* = 0.011) and marginally larger than that of near-threshold scenarios (M = 17.18, SE = 1.53, *p* = 0.075). Contrary to the hypothesis, there was no main effect of the GBA method. The observed effect size for the GBA method was partial η^2^ of 0.056, which translates to an effect size f of 0.24 and is smaller than our expectation of 0.3 in the a priori power analysis.

### 3.3. Subjective Usability

**Figure 8:**
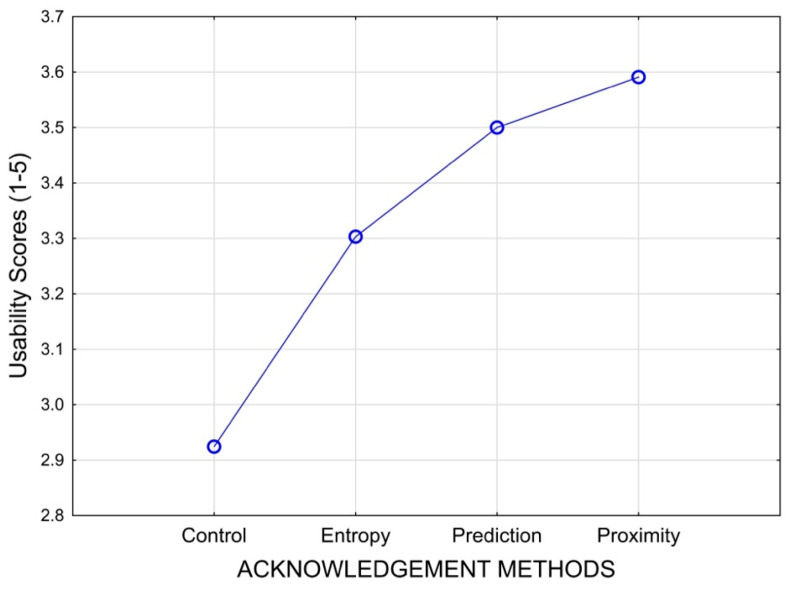
Mean plot of GBA methods’ main effect on usability ratings, F(3,63) = 3.745, *p* = 0.015, partial η^2^ = 0.151. Confidence intervals were omitted to avoid statistical inference with repeated measure ANOVA.

The usability scale showed good internal consistency across the three items (Cronbach α = 0.83). The error residuals were not normally distributed (Shapiro–Wilk test W = 0.017, *p* = 0.02), but the kurtosis and skewness were −0.39 and −0.31 within the range between 1 and -1, respectively; therefore, the ANOVA should remain robust against the normality assumption [87,88]. The sphericity assumption was satisfied under Mauchly’s criterion (*X*^2^(3) = 3.84, *p* = 0.57). The one-way ANOVA revealed a significant effect for GBA method on usability scores, F(3,63) = 3.745, *p* = 0.015, partial η^2^ = 0.151 (Figure 8). Planned contrast analyses revealed that usability ratings were lower for no GBA (control condition) than GBA methods collectively, F(2,22) = 4.57, *p* = 0.021.

### 3.4. Associations with Alarm Prediction Performance

A Chi-squared test was conducted to examine how well the criteria of different GBA methods (fixations on AOI prior to alarm and parameter behaviors) represented explicit alarm prediction and revealed a significant association between alarm acknowledgement and prediction hits in a trial, *X*^2^ (1, *N* = 432) = 23.802, *p* < 0.001. The likelihood of prediction hits was significantly higher in trials where the participant acknowledged the alarm through the GBA methods compared to trials where the participant did not, OR = 3.877, 95% CI 2.25–6.68, *p* < 0.05. Table 5 also shows that alarm acknowledgements through fixations occurred infrequently (only 65 out of 432 trials). This statistical analysis omitted 144 trials of the no GBA condition that precluded any acknowledgement.

Two Chi-squared tests were performed to determine whether GBA methods and scenario types were associated with prediction hits or misses. The Chi-square tests only revealed a relationship between prediction hits or misses and scenario types (*X*^2^ (2, *N* = 576) = 10.567, *p* = 0.01 (Table 6) but no association with GBA methods, *X*^2^ (3, *N* = 576) = 0.874, *p* = 0.831. After Bonferroni adjustment to the error rate for post hoc testing, the likelihood of alarm prediction hit was significantly higher in near-threshold than trending scenarios, OR = 2.26, 95% CI 2.25–6.68, *p* = 0.018.

A Chi-squared test was conducted to determine whether alarm prediction hits were contingent on GBA methods and scenario types, but it did not reveal a significant association between GBA methods and scenario types in prediction hits (*X*^2^ (6, *N* = 91) = 1.561, *p* = 0.955).

### 3.5. Association with Acknowledgement Frequency/Usage

Chi-squared tests revealed a marginally significant association of alarm acknowledgements (i.e., implicit usage of GBA) with the GBA method, *X*^2^ (2, *N* = 432) = 5.494, *p* = 0.064 (Table 7), and a significant association with scenario types, *X*^2^ (2, *N* = 432) = 30.147, *p <* 0.001 (Table 8). After Bonferroni adjustment to the error rate for post hoc testing, the likelihood of alarm acknowledgement through gaze was significantly higher in fluctuation than near-threshold scenarios, OR = 3.50, 95% CI 2.07–5.94, *p* < 0.01, and trending scenarios, OR = 3.08, 95% CI 1.84–5.16, *p* < 0.01. The statistical tests on alarm acknowledgements omitted the 144 trials of no GBA condition that precluded any acknowledgement.

A Chi-squared test revealed a significant association between GBA methods and scenario types in the number of alarm acknowledgements through gaze (*X*^2^ (4, *N* = 125) = 26.55, *p* < 0.001; Table 9). That is, the likelihood of alarm acknowledgements occurring was dependent on the interaction of the scenario type (i.e., parameter behavior) and GBA methods. However, the hypothesized pattern that most acknowledgments occurred in pairs, including the entropy method with fluctuation scenarios, the prediction method with trending scenarios, and the proximity method with near-threshold scenarios, was not apparent.

The analysis of the association of alarm prediction performance involved a series of five Chi-squared tests that could inflate the Type 1 error rate even though we presented a priori hypotheses. However, we do not foresee significant risk to the results, as the *p*-values are below 0.01, except for a marginally significant association between alarm acknowledgements and the GBA method. Readers should be cognizant of this minor risk to the Type 1 error rate.

## 4. Discussion

This research aims to advance the user-centric approach to alarm management by developing and evaluating three GBA methods that inferred alarm anticipation and modulated visual and auditory signals of alarms to better match attentional demand between parallel tasks. The application of eye tracking to infer alarm expectancy is novel, and the study results represent the initial empirical evidence on alarm management using an eye-fixation metric.

### 4.1. Impact of Gaze-Based Acknowledgement on Parallel Tasks

The study presents moderate support for our novel user-centric approach of employing eye trackers to infer alarm expectancy and moderate alarm signals. The GBA methods showed better usability ratings over no GBA, but the ANOVA results did not reveal any performance benefits for the parameter control task. The Chi-squared results indicate that alarm acknowledgement tended to precede alarm prediction hits, confirming that eye fixation is a reasonable measure for inferring alarm anticipation. While the benefits are not absolute, this study illustrates that GBA represents a promising method for enhancing alarm management from a user-centric perspective.

Two explanations are provided for the lack of significant effect of the GBA method on control task performance. The first explanation is that only the alarm monitoring task involved auditory alerts, which had to compete for auditory resources if the parameter control task also included auditory alerts (referring to multiple resource theory [89]). That is, muting auditory signals of alarms may have a bigger impact on parallel task performance if both tasks contain auditory signals. Such task designs would also have been more representative of many current work settings, such as control rooms of nuclear power plants. In other words, control task performance may be less sensitive to the effect of the GBA methods than expected because the parallel tasks are not competing for identical pools of cognitive resources.

The second and related explanation is that the participants focused on the parameter control task more often than the alarm monitoring task, as evidenced by 65% of the trials having alarm prediction misses and 72% of the trials having no alarm acknowledgement when provided GBA methods (Table 4). From the perspective of executive control in the dual-task paradigm [90], once the participants engaged in the parameter control task (which intrinsically required user input about every ten seconds), there is zero salience for switching back to the alarm monitoring task until an alarm is sounded (which intrinsically required user input about every 70 s). Because of the greater attention demand for the parameter control task and the absence of alerts to switch to the alarm monitoring task, the participants likely over-engaged in the parameter control task, resulting in performance plateaus irrespective of GBA methods.

In summary, the effectiveness of GBA methods is dependent on the characteristics of tasks competing against alarm monitoring. We attribute the lack of significant effect on task performance to the parallel task design in this study, which likely masked some GBA benefits. In this study, the participants paid imbalanced or unequal attention between the two parallel tasks. Specifically, the parameter control task outcompeted the alarm monitoring task for attention, likely leading to a lack of significance for the parameter control task’s performance. In a real-world and ecologically valid environment, the outcome could be different. We believe GBA would be more effective in supporting real-world control room operators, who chiefly perform supervisory rather than manual control. That is, the operators would be more occupied with monitoring multiple parameters and making discrete control inputs (e.g., shutting a valve), so the parallel tasks in the real world would likely compete for the same level of attention and the same pools of cognitive resources. While the parallel task design may have reduced the power of detecting performance benefits, the results reinforce that the GBA methods permit inattentive operators to receive normal alarm indications, and it has high usability as a method for user-centric alarm management.

### 4.2. Compatibility Between Visual Sampling and Parameter Behaviors for Gaze-Based Management

Parameter behavior of the impending alarm exerted a consistent effect on parameter control task performance, alarm prediction, and acknowledgement frequency/usage, providing confirming evidence that compatibility between visual sampling and parameter behaviors is critical for GBA methods for user-centric alarm management. The fluctuation scenario type is particularly notable, because this parameter behavior induced the most alarm acknowledgements (i.e., significantly higher odds ratio of alarm acknowledgements by gaze compared to other scenario types) but did not yield the best alarm prediction performance relative to other scenario types (i.e., it did not have a significantly higher odds ratio of prediction hits to other scenario types). We attribute this outcome to the trendline variability of the fluctuation scenarios making alarm trajectory estimation more difficult. Another potential explanation is that the graph of a fluctuating parameter attracted greater attention of peripheral vision, which led to more fixations irrespective of any substantial awareness of an impending alarm.

The association of the fluctuation scenario type with alarm acknowledgement (by gaze) but not prediction hits also reflect the result that alarm acknowledgements by gaze did not guarantee alarm prediction hit (Table 4). Specifically, prediction misses remained high even when alarm acknowledgements by gaze occurred, suggesting that eye fixations on the target AOIs, beyond the defined threshold, could have other interpretations besides alarm expectation. Holmqvist et al. [52] cited research that interprets higher fixations as uncertainty of what a person observed, despite previous research indicating that vigilant monitoring would increase operator expectancy and nuisance alarms [44,45]. In a study employing eye fixations as the basis to alert pilots, Schwerd and Schulte [91] discovered a similar design limitation in their adaptive display that falsely assumed fixations to be related to perception in the same manner across all situations. A potential resolution to such ambiguity is to set an upper fixation threshold, which would indicate uncertainty about parameter behavior attracting high visual attention. None of the GBA methods in this study included such an upper fixation limit. Personalization of the fixation thresholds may be necessary because expertise and experience can play a role in gaze behaviors, as demonstrated in a recent study that discovered changes in look ahead fixations (i.e., early eye disengagement from the target) with task complexity and repetitions [92].

### 4.3. Relevance to the Alarm Literature

This work contributes to research on user sensing for alarm management, specifically in proposing and evaluating the use of eye trackers as part of the user-centric approach for optimizing alarms. While alarms serve “as the primary communicator between the automated system and the operator” [93], current alarm design constrains the operator to wait for the alarm sound, rather than when it is first noticed. This leads to circumstances where operators react twice: once when they first anticipate the alarm, and then again when manually acknowledging the alarm. Unless real-time sensing is used to infer the operator’s understanding, the user interface or alarm management system will not be able to maximize alarm informativeness and eliminate all nuisance alarms. This is why using eye trackers for real-time measurements of operator awareness of alarms and modulating the salience of visual and auditory signals present promise.

This study reveals potential factors for estimating alarm anticipation (i.e., fixations on target AOI and parameter behaviors) while recognizing that more sophisticated design requirements of GBA are needed to fully calibrate for alarms. An operator’s awareness of parameters reaching alarm status has more granular states of understanding than being aware and confused, as hypothesized before this study. Lees and Lee [46] distinguished alarm predictability from performance utility, as researchers have observed operators following an alarm and monitoring the situation for a period before acknowledgment and silencing the alarm [94]. In these cases, the operators may be diagnosing the alarms with both awareness and confusion of parameter behaviors. The nature of having knowledge about alarms during abnormal situations is complex, as operators might be seeking to monitor parameter behaviors in contrast to their internal expectations of the plant’s state in order to affirm alarm utility [4]. The cognitive complexity of re-interpreting parameter behaviors with respect to alarms stands in contrast to our design basis for the alarm acknowledgement methods—the assumption that the operator can correctly predict impending alarms given sufficient visual attention even for a simple simulated process. The study’s results thus highlight another research gap in the literature, which currently does not contain any taxonomy or scale to define the types and levels of operator knowledge about an alarm. This research inquiry is vital to resolve nuisance alarms from a user-centric perspective.

This study also highlights a need for better design guidance in exploiting eye tracking data to a much greater degree. Hvelpund [95] recounted that mean fixation duration can differ by fifty milliseconds depending on whether the task requires visual search or scene perception. By extension of such findings, GBA of alarms could improve if fixation or other gaze metric requirements differ by parameter behavior, which has also been recognized in [91]. Additional gaze metrics, like fixation rates, scan paths, body orientation, proximity, and other physiological indicators (e.g., electroencephalogram [EEG] and heart rate variability [HRV]) in support of multi-modal sensing, could also provide incremental validity to inferencing alarm expectation (e.g., [96]). The literature currently lacks clear guidance on utilizing multiple gaze metrics jointly as leverage for an adaptive user interface design to preserve selective attention in human–computer interactions, such as Attentive User Interfaces (AUI) and gaze-contingent displays [97,98,99,100].

### 4.4. Limitations

This study has four major limitations. First, the sample size might need to be larger than 24 participants given the hindsight that the parameter control task unexpectedly and substantially competed for attention against the alarm monitoring task. Given the evidence supporting a large attentional disparity towards the parameter control task over monitoring alarms, the effect size of GBA on control task performance is probably smaller than the estimate used in our power analysis for determining sample size. Future power analysis for studies on gaze GBA would need to moderate effect size estimation with respect to the design of parallel tasks.

Second, although the general design principle for each GBA method should be applicable to many real-world applications, the exact mathematical formulation for modeling parameter behaviors and setting thresholds require more sophistication for real plants. For example, second-order polynomial regression of the past 15 s of data for predicting the next 3 s of parameter values may only be sufficient for the parameters in this simulator. This study also did not investigate how humans would respond with GBA for alarms that are not perfectly reliable or with varying false alarm rates.

Third, this research presents an early investigation of gaze interaction that was tested with college students performing laboratory tasks. The exact utility of the GBA methods could change with expertise or for plant operators. Moreover, GBA methods may improve when accounting for the unique mental models and strategies of professional operators. The findings in this study should be taken to inform a novel complementary design approach rather than observations to be expected with experienced operators controlling complex processing plants.

Finally, the study did not replicate the typical time length of a work shift or the complexity of work tasks. As fatigue typically accrues over a shift, minimizing nuisance stimuli might have some accumulated effects undetectable in this study. The two parallel tasks were built on two simple simulations representing a small set of operator activities and industrial processes. Operators often need to work with maintenance, engineering, and management in addition to controlling some key parameters manually and monitoring alarms. The complexity of work settings can also influence the effectiveness of GBA methods. More representative and diverse tasks resembling real-world environments and shifts should be designed for future research to assess possible benefits in practical settings.

## 5. Conclusions

This research contributes the first empirical evidence on using eye tracking for assessing alarm anticipation and GBA for user-centric alarm management. This research study evaluated three GBA methods using non-intrusive measurements of eye gaze to infer alarm anticipation and moderate visual and auditory signals of alarm (i.e., saliency) to minimize disruption between parallel tasks. The study’s results revealed that usability ratings were better with than without GBA methods (F(3,63) = 3.745, *p* = 0.015, partial η^2^ = 0.151). Furthermore, alarm acknowledgements by gaze tended to co-occur with alarm prediction hits (*X*^2^ (1, *N* = 432) = 23.802, *p* < 0.001, OR = 3.877, 95% CI 2.25–6.68, *p* < 0.05), indicating that eye fixation could be a reasonable measure for inferring alarm anticipation. Finally, parallel task performance and alarm acknowledgements by gaze were contingent on parameter behaviors (F(2,46) = 5.075, *p* = 0.011, partial η^2^ = 0.184; *X*^2^ (2, *N* = 432) = 30.147, *p <* 0.001), highlighting the need to account for system behaviors with respect to human awareness in reducing nuisance alarms. Taken together, the study indicates that eye tracking technology can enable the estimation of alarm anticipation for GBA to complement existing alarm management strategies.

Subsequent studies could adjust the task design to make the two parallel tasks more comparable in terms of auditory perception or further explore the mechanism through which this difference affects the results. Additional indicators, such as cognitive load or fatigue, could be included to give more insight into user experience. Future work should also investigate any interaction between expertise and parameter behaviors, as eye gaze patterns are often different between exerts and novices [53,64,101], to strengthen the estimation of alarm expectancy. With further understanding of eye–gaze behaviors for inferring alarm awareness, machine learning on eye tracking and biometric data should be investigated to optimize the inference of cognitive states, which has been successful in other applications [102,103]. Although this study indicates that GBA methods have usability advantages, other practical factors, such as system stability and misjudgment rate, should be incorporated into determining the level of appropriate risk tolerance against acknowledging alarms incorrectly or unintentionally. This may include refining the design to “unacknowledged” of some acknowledged alarms by gaze after a short period of time if safety issues persist or operators are deemed unaware of the alarms. Finally, future research should examine the sensitivity and operability of GBA in conjunction with existing alarm systems holistically. These future research studies must also address ecological validity by recruiting professional operators completing tasks in high-fidelity simulators to assess generalization of the study findings collected from college students operating a low-fidelity simulator.

Given extensive opportunities for refining the design, the implementation of GBA of alarms would likely require the following efforts First, GBA will need to improve its inference accuracy by improving the use of gaze data, coupled with other biometric sensors, and by developing advanced data fusion algorithms. Second, the user interface design will need to support the recovery of any errors in the inference of alarm expectancy, including re-issuing salient alarms in the absence of operator response to gaze-acknowledged alarms. Finally, a safety validation study is necessary to assess whether operators are responding to alarms more appropriately, as critical process information could be overlooked due to alarms lacking salience or too many salient alarms. While further refinement and validation are necessary for GBA, this study presents the first evidence that eye trackers can play an important role in next-generation alarm management systems for inferring operator awareness and reducing nuisance alarms.

## Figures and Tables

**Table 1:** Experimental measures.

Category	Measure
Control Task Performance	Time out of permissible range for the feedwater tank level in a trial
Alarm Prediction Performance	Prediction hits and misses in a trial
Acknowledgement Usage	Gaze-based acknowledgment (occurred) in a trial
Usability	3-item subjective questionnaire (Table 2)

**Table 2:** Alarm prediction performance variables in terms of signal detection theory.

Signal Detection Theory	Alarm Prediction Performance
Miss	No click on target AOI within 4 s prior to an alarm
False Alarm	Clicks on target AOI, inaccurate time (t > 4 s or time < 1 s); clicks on incorrect AOI any time
Hit	Clicks on target AOI with alarm incurring within 4 s prior to alarm
Correct Rejection	Not Applicable all trials have an alarm present

AOI denotes areas of interest.

**Table 3:** Subjective usability questionnaire.

No.	Statement (1 = Strongly Disagree, 5 = Strongly Agree)
1	Gaze-based acknowledgment anticipated my needs.
2	Gaze-based acknowledgement was correct in assuming I anticipated the alarm.
3	Gaze-based acknowledgement was not effective in minimizing alarms I was aware of.

**Table 4:** ANOVA of time out of range for feedwater tank level (i.e., parameter control task performance) by GBA method and scenario type. * Indicates a *p*-value < 0.05, the threshold of statistical significance.

Effects	Eff df	Error df	F-Value	*p*-Value	Partial η^2^
Gaze-Based Acknowledgement Method	3	69	1.357	0.263	0.056
Scenario Type	2	46	5.075	0.011 *	0.181
Gaze-Based Acknowledgement Method × Scenario Type	6	138	1.597	0.152	0.065

Eff df = degrees of freedom for the effect; Error df = degrees of freedom for the error term.

**Table 5:** A contingency table of alarm prediction hits and misses by alarm acknowledgement through gaze (i.e., implicit usage), which illustrates the level of accuracy any GBA method has, representing participants’ explicit alarm prediction.

		Trial Count of Alarm Prediction		
		Hit	Miss	Total
Acknowledgement Usage	Acknowledged	36	29	65
	Not Acknowledged	89	278	367
	Total	125	307	432

**Table 6:** A contingency table of alarm prediction hits and misses by scenario type, which illustrates the relationship between prediction accuracy and the parameter characteristics of the impending alarm.

		Trial Count of Alarm Prediction		
		Hit	Miss	Total
Scenario Types	Fluctuation	28	164	192
	Near-threshold	43	149	192
	Trending	20	172	192
	Total	91	485	576

**Table 7:** A contingency table of gaze-based acknowledgement frequency (i.e., implicit usage) by acknowledgement method administered to indicate whether the methods received equal usage.

		Trial Count of Alarm Acknowledgement by Gaze		
		Acknowledged	Not Acknowledged	Total
Gaze-Based Acknowledgement Method	Entropy	33	111	144
	Prediction	41	103	144
	Proximity	51	93	144
	Total	125	307	432

**Table 8:** A contingency table of gaze-based acknowledgement frequency (i.e., implicit usage) by scenario type to illustrate whether the parameter characteristics of the impending alarm were acknowledged equally.

		Trial Count of Alarm Acknowledgement (by Gaze)		
		Acknowledged	Not Acknowledged	Total
Scenario Types	Fluctuation	66	78	144
	Near-threshold	28	116	144
	Trending	31	113	144
	Total	125	307	432

**Table 9:** A contingency table illustrating whether alarm acknowledgement frequency (i.e., implicit usage) favored the GBA method designed for the scenario type (entropy with fluctuation scenarios; prediction with trending scenarios; proximity with near-threshold scenarios).

		Count of Trials Acknowledged Across Scenario Type			
		Fluctuation	Near-Threshold	Trending	Total
Acknowledgement Method	Entropy	28	0	5	33
	Prediction	18	8	15	41
	Proximity	20	20	11	51
	Total	66	28	31	125

## Data Availability

The raw data supporting the conclusions of this article will be made available by the authors on request.

## Abbreviations

The following abbreviations are used in this manuscript:

AOI	Area of interest
EEG	Electroencephalogram
GBA	Gaze-based acknowledgement
HRV	Heart rate variability
